# Bioconversion of Rebaudioside I from Rebaudioside A

**DOI:** 10.3390/molecules191117345

**Published:** 2014-10-28

**Authors:** Indra Prakash, Cynthia Bunders, Krishna P. Devkota, Romila D. Charan, Catherine Ramirez, Tara M. Snyder, Christopher Priedemann, Avetik Markosyan, Cyrille Jarrin, Robert Ter Halle

**Affiliations:** 1 The Coca-Cola Company, Atlanta, GA 30313, USA; E-Mail: cbunders@coca-cola.com; 2 AMRI-Albany, Analytical Development, Albany, NY 12212, USA; E-Mails: Krishna.Devkota@amriglobal.com (K.P.D.); Romila.Charan@amriglobal.com (R.D.C.); Catherine.Ramirez@amriglobal.com (C.R.); Tara.Snyder@amriglobal.com (T.M.S.); Christopher.Priedemann@amriglobal.com (C.P.); 3 PureCircle Limited, Lengkuk Teknologi, Bandar Enstek 71760 Malaysia; E-Mail: avetik@purecircle.com; 4 Libragen SA, 3 Rue des Satellites, Toulouse 31400, France; E-Mails: c.jarrin@libragen.com (C.J.); r.terhalle@libragen.com (R.T.H.)

**Keywords:** *Stevia rebaudiana*, structure characterization, bioconversion, rebaudioside I, rebaudioside A

## Abstract

To supply the increasing demand of natural high potency sweeteners to reduce the calories in food and beverages, we have looked to steviol glycosides. In this work we report the bioconversion of rebaudioside A to rebaudioside I using a glucosyltransferase enzyme. This bioconversion reaction adds one sugar unit with a 1→3 linkage. We utilized 1D and 2D NMR spectroscopy (^1^H, ^13^C, COSY, HSQC-DEPT, HMBC, 1D TOCSY and NOESY) and mass spectral data to fully characterize rebaudioside I.

## 1. Introduction

In the past decade the food industry has dedicated concerted efforts to develop natural non-caloric high potency sweeteners. The demand for alternative sweeteners comes from the overwhelming consumer preference for taste of sugar, but without calories. The food industry has heavily invested in the discovery of novel natural steviol glycosides that have the ability to sweeten products and provide no caloric content. Steviol glycosides are most commonly isolated from *Stevia rebaudiana* Bertoni, a perennial shrub of the *Asteraceae* (*Compositae*) family native to Paraguay and Brazil [1]. Isolation of steviol glycosides from *S. rebaudiana* leaves over the past thirty years have uncovered the wide variety of their molecular complexity [2,3,4,5,6,7,8,9,10,11]. Most recently, Ibrahim *et al.* isolated two novel diterpene glycosides and five known glycosides from a commercial extract of *S. rebaudiana* [12]. Steviolbioside, stevioside, rebaudioside A–F, dulcoside A and rubusoside are the most abundant steviol glycosides found in the leaves of *S.* *rebaudiana* [13].

**Figure 1:**
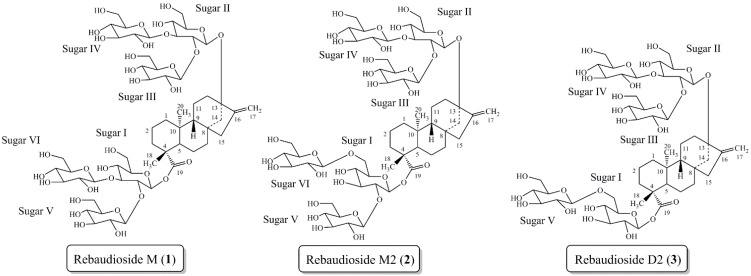
Steviol glycoside rebaudioside M (**1**) isolated from *S. rebaudiana* Bertoni. Steviol glycosides rebaudioside M2 (**2**) and rebaudioside D2 (**3**) isolated from the bioconversion reaction of rebaudioside A to rebaudioside D.

Recently we reported the isolation, full characterization, and sensory properties of rebaudioside M (**1**) from *S. rebaudiana* Bertoni (Figure 1) [14,15]. Rebaudioside M is approximately 200–350 times as potent as sucrose [15]. Sensory evaluations determined rebaudioside M’s taste profile to be a clean, sweet taste, with a slightly bitter and licorice-like aftertaste. This work illustrated that the configuration of glycosylation (number of sugar units and the sugar linkages) influences the sweet taste profile of steviol glycoside molecules.

Due to the relative low abundance of certain sweet steviol glycosides in plant material, numerous organizations have invested in biotechnology to produce larger quantities of desired steviol glycosides [16,17,18,19,20,21,22,23]. Most recently we have pursued the bioconversion reaction of rebaudioside A to rebaudioside D with UGT (UDP-glycosyltransferase) enzymes. This bioconversion reaction allowed for the isolation and characterization of two novel steviol glycosides, rebaudioside M2 (**2**) and rebaudioside D2 (**3**) (Figure 1) [24,25]. Both molecules contain a 1→6 sugar linkage, which is rarely found in the steviol glycoside family.

**Figure 2:**
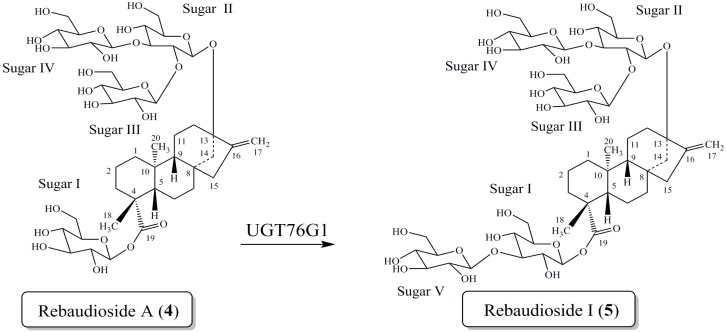
Bioconversion of rebaudioside A (**4**) to rebaudioside I (**5**).

**Figure 3:**
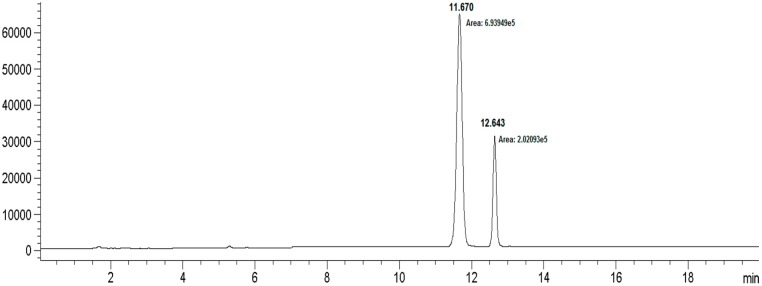
HPLC chromatogram for the bioconversion of rebaudioside I from rebaudioside A.

Based on previous experiences with the bioconversion reactions we investigated the bioconversion of rebaudioside A (**4**) to rebaudioside I (**5**) (Figure 2). The bioconversion reaction was analyzed by HPLC with reference standards. Rebaudioside I, a natural non-claoric sweetener, was produced with a 22.5% yield (135 mg) (based on total area percent of HPLC-MS SIM chromatography) (Figure 3). We report herein the isolation and full characterization of rebaudioside I, determined by 1D and 2D NMR experiments together with mass spectral data. Ohta *et al.* first reported the isolation of rebaudioside I in *S. rebaudiana* Morita, although full spectral assignment was not reported [9,26].

**Table:** 

Compound	Retention Time	MW	Peak Area
Rebaudioside I (**5**)	11.670	1129.16	693,949
Rebaudioside A (**4**)	12.643	967.02	202,093

## 2. Results and Discussion

Rebaudioside I (**5**) was isolated as a white solid and accurate mass measurement using High Resolution Mass Spectrometry (HRMS) provided an exact mass *m/z* of 1127.4741, [M−H]^−^ in its negative ESI-TOF mass spectrum, corresponding to the molecular formula C_50_H_79_O_28_ (see Supplementary Information). The MS/MS spectrum of **5**, selecting the [M−H]^−^ ion at *m/z* 1127.4 for fragmentation, indicated loss of two sugar units at *m/z* 803.5301, however it did not show additional fragmentations with a collision energy of 30 V. When a higher collision energy was applied (60 V), the parent ion was not observed, but sequential loss of three sugar units at *m/z* 641.4488, 479.3897, and 317.3023 were observed from *m/z* 803.5301.

Having confirmed the molecular weight of compound **5**, a series of 1D and 2D NMR experiments were performed to allow for the full assignment of rebaudioside I. Our initial ^1^H-NMR spectrum was acquired at 300 K, where one of the anomeric protons was completely obscured by the water resonance. Therefore, ^1^H-NMR spectrum of the sample was acquired at lower temperature (292 K), to shift out the water resonance, and at this temperature all anomeric protons were sufficiently resolved (Supplementary Information). Thus, all other NMR data of **5** was acquired at 292 K.

**Figure 4:**
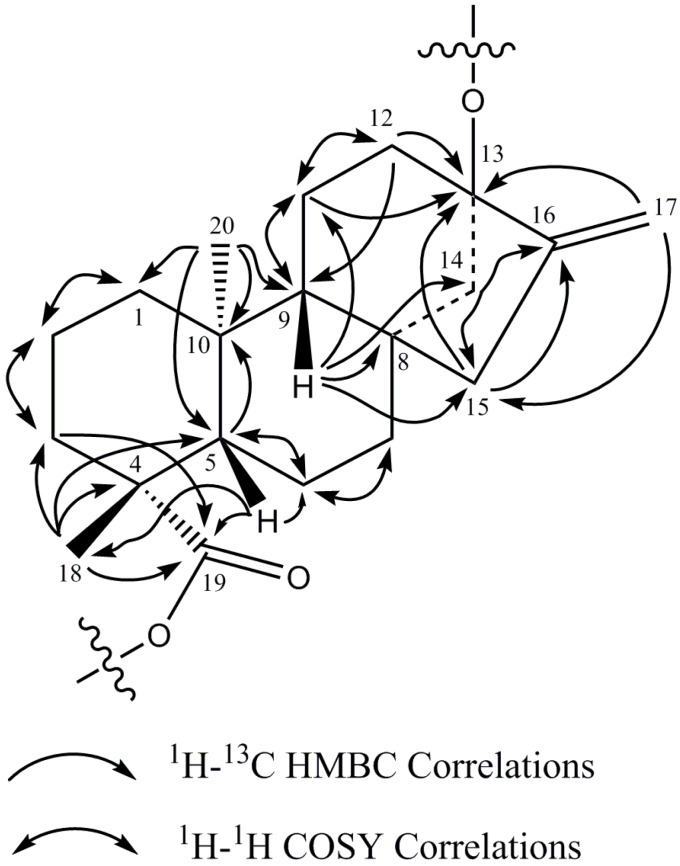
^1^H-^1^H COSY and ^1^H-^13^C HMBC correlations of the diterpene core of rebaudioside I (5).

The ^1^H-NMR spectrum and the HSQC-DEPT data of rebaudioside I (**5**) indicated the existence of two methyl singlets at δ 1.22 (C-18) and 1.26 (C-20), two olefinic proton singlets corresponding to an exocyclic double bond at δ_H_ 5.02 and at 5.67 (C-17), nine methylene and two methine protons between δ_H_ 0.74–2.59 characteristic for the *ent*-kaurane diterpenoid isolated from other *Stevia* extracts [27,28]. The *ent-*kaurane diterpenoid aglycone central core was supported by ^1^H-^1^H COSY and ^1^H-^13^C HMBC correlations shown in Figure 4. The complete ^1^H and ^13^C assignments of the central diterpene core are provided in Table 1 (positions 1–20).

Correlations observed in the NOESY spectrum were used to assign the relative stereochemistry of the central diterpene core. In the NOESY spectrum, NOE correlations were observed between H-14 and H-20 indicating that H-14 and H-20 are on the same face of the rings. Similarly, NOE correlations were observed between H-9 and H-5 as well as H-5 and H-18. NOE correlations between H-9 and H-14 were not observed. The NOESY data thus indicate that H-5, H-9 and H-18 were on the opposite face of the rings compared to H-14 and H-20. These data thus indicate that the relative stereochemistry in the central core was retained during the glycosylation step.

**Table 1:** ^1^H- and ^13^C-NMR (500 and 150 MHz, pyridine-*d*_5_ + TMS), assignments of the rebaudioside I (**5**).

Sugar	Position	^1^H-NMR	^13^C-NMR
	1	0.74 t (11.6), 1.75 m	40.7
	2	1.44 m, 2.20 m	19.4
	3	1.02 m, 2.35 m	38.5
	4	---	44.0
	5	1.03 m	57.2
	6	1.90 m, 2.33 m	22.2
	7	1.29 m, 1.31 m	41.7
	8	---	42.3
	9	0.88 d (6.3)	54.1
	10	---	39.8
	11	1.67 m, 1.70 m	20.5
	12	1.98 m, 2.28 m	37.3
	13	---	86.7
	14	1.78 m, 2.59 d (11.9)	44.3
	15	2.04 brs	47.6
	16	---	154.0
	17	5.02 s, 5.67 s	104.8
	18	1.22 s	28.4
	19	---	176.9
	20	1.26 s	15.7
I	1'	6.14 d (8.2)	95.3
	2'	4.18 m	72.5
	3'	4.27 m	89.4
	4'	4.25 m	69.2
	5'	3.93 m	78.2–78.8 ^†^
	6'	4.27 m, 4.37 m	61.7
II	1''	5.06 d (7.9)	98.0
	2''	4.35 m	80.6
	3''	4.20 m	87.5
	4''	3.97 m	70.1
	5''	3.80 m	77.6
	6''	4.18 m, 4.49 m	62.5
III	1'''	5.57 d (7.7)	104.6
	2'''	4.21 m	76.3
	3'''	4.27 m	78.2–78.6 ^†^
	4'''	4.25 m	72.1
	5'''	3.94 m	78.2–78.8 ^†^
	6'''	4.41 m, 4.53 m	63.1
IV	1''''	5.38 d (7.9)	104.7
	2''''	4.01 m	75.3 or 75.5
	3''''	4.28 m	78.2–78.6 ^†^
	4''''	4.11 m	72.1
	5''''	4.13 m	78.2–78.6 ^†^
	6''''	4.25 m, 4.58 m	62.3 or 62.4
V	1'''''	5.29 d (7.9)	105.0
	2'''''	4.04 m	75.3 or 75.5
	3'''''	4.27 m	78.2–78.6 ^†^
	4'''''	4.12 m	71.5 or 71.6
	5'''''	4.05 m	78.5 or 78.6 ^†^
	6'''''	4.26 m, 4.56 m	62.3 or 62.4

^†^ Five carbon resonances in the range of 78.2–78.8 (78.16, 78.47, 78.50, 78.55, and 78.77), hence chemical shift could not be unequivocally assigned.

The breakdown of the ^1^H-^13^C HSQC-DEPT data for **5** confirmed the presence of five anomeric protons. All five anomeric protons were resolved in the spectra acquired at 292 K at δ_H_ 6.14 (δ_C_ 95.3), 5.57 (δ_C_ 104.6), 5.38 (δ_C_ 104.7), 5.29 (δ_C_ 105.0), and 5.06 (δ_C_ 98.0) (Table 1). Additionally, all five anomeric protons had large couplings (7.7–8.2 Hz) indicating that they had β-configurations. The anomeric proton observed at δ_H_ 6.14 showed an HMBC correlation to C-19 (δ_C_ 176.9) which indicated that it corresponds to the anomeric proton of Glc_I_. Similarly, the anomeric proton observed at δ_H_ 5.06 showed an HMBC correlation to C-13 (δ_C_ 86.7) allowing it to be assigned as the anomeric proton of Glc_II_ (Figure 4).

Additional analysis of the 1D and 2D NMR data allowed the assignment of the remaining three sugars in **5**. The relatively downfield chemical shift of C-3' (δ_C_ 89.4) in sugar I suggested the presence of a sugar substituent at C-3' of sugar I. Long range ^1^H-^13^C correlations observed in the HMBC experiment from the anomeric proton observed at δ_H_ 5.29 (H-1''''') to the carbon at δ_C_ 89.4 (C-3') and from H-3' at δ_H_ 4.27 to an anomeric carbon at δ_C_ 105.0 (C-1''''') confirmed the substitution at C-3' in sugar I.

The remaining two glucose moieties were assigned in a similar manner. The relatively downfield chemical shift of C-2'' (δ_C_ 80.6) and C-3'' (δ_C_ 87.5) in sugar II suggested a 2,3-branched-d-glucotriosyl substituent at C-13. Long range ^1^H-^13^C correlations observed in the HMBC experiment from the anomeric proton observed at δ_H_ 5.57 (H-1''') to the carbon at δ_C_ 80.6 (C-2'') and from H-2'' at δ_H_ 4.35 to an anomeric carbon at δ_C_ 104.6 (C-1''') confirmed the presence of a 1**→**2 sugar linkage between sugar III and sugar II. Similarly, the sugar substituent at C-3'' in sugar II was also corroborated by HMBC correlations observed from the anomeric proton at δ_H_ 5.38 (H-1'''') to the carbon at δ_C_ 87.5 (C-3'') and from H-3'' at δ_H_ 4.20 to the anomeric carbon (δ_C_ 104.7) of sugar IV confirmed the presence of a 1**→**3 sugar linkage between sugar IV and sugar II.

**Figure 5:**
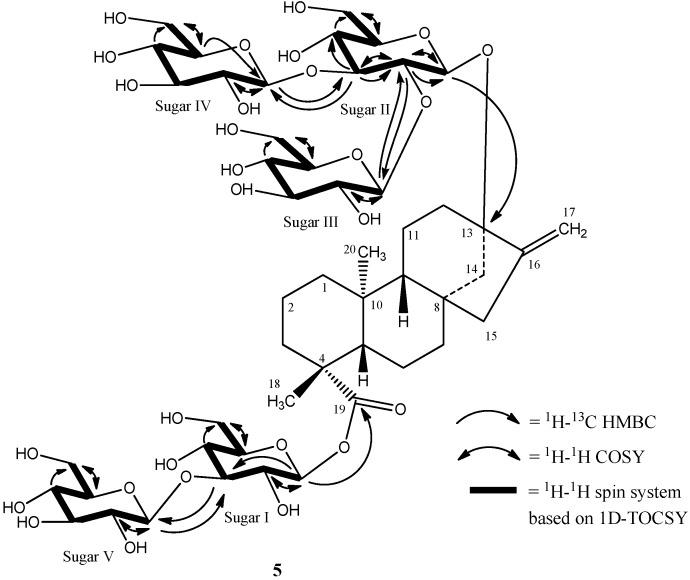
Key COSY, HMBC, and 1D-TOCSY correlations of rebaudioside I (**5**).

The complete ^1^H and ^13^C assignments for the glycoside at C-13 and C-19 were made on the basis of COSY, HSQC-DEPT, HMBC, and 1D-TOCSY data and are provided in Table 1. A summary of the key HMBC, COSY, and 1D-TOCSY correlations used to assign the glycoside are provided in Figure 5.Thus the structure of rebaudioside I (**5**), was confirmed as (13-[(2-*O*-β-d-glucopyranosyl-3-*O*-β-d-glucopyranosyl)-β-d-glucopyranosyl)oxy] *ent*-kaur-16-en-19-oic acid-(3-*O*-β-d-glucopyranosyl)-β-d-glucopyranosyl) ester].

## 3. Experimental Section

### 3.1. General Experimental Procedures for Rebaudioside I (**5**)

#### 3.1.1. Isolation of Rebaudioside I

Preliminary HPLC analyses of samples were performed using a Waters 2695 Alliance System (Waters Corp., Milford, MA, USA) equipped with a Waters 2996 Photodiode Array (PDA, Waters Corp.) and Dionex Corona Charged Aerosol (CAD Plus, Dionex, Sunnyvale, CA, USA) detectors by the following method: Phenomenex Synergi Hydro-RP, 4.6 × 250 mm, 4 µm (p/n 00G-4375-E0); Column Temp: 55 °C; Mobile Phase A: 0.00284% NH_4_OAc and 0.0116% HOAc in water; Mobile Phase B: Acetonitrile (MeCN); Flow Rate: 1.0 mL/min; Injection volume: 10 µL. Detection was by UV (210 nm) and CAD. Gradient: 0–8.5 min (75A:25B), 10 min (71A:29B), 16.5 min (70A:30B), 18.5–24.5 min (66A:34B), 26.5–29.0 min (48A:52B), 31–37 min (30A:70B), 38 min (75A:25B).

HPLC Isolation: The purification was performed in two chromatographic steps. The first method used a Waters Symmetry Shield RP18 (30 × 150 mm, 7 µm, p/n WAT248000) column with isocratic mobile phase conditions of 80:20 water/methanol (MeOH). Flow rate was maintained at 45 mL/min and injection load was 180 mg. Detector wavelength was set at 210 nm. The second chromatographic method used a Waters Atlantis dC18 (30 × 100 mm, 5 µm, p/n 186001375) column with isocratic mobile phase conditions of 80:20 water/MeCN. Flow rate was maintained at 45 mL/min and detector wavelength was set at 210 nm. The analyses of fractions were performed using a Waters Atlantis dC18 (4.6 × 150 mm, 5 µm, p/n 186001342) column; Mobile Phase A: water; Mobile Phase B: MeCN; Flow Rate: 1 mL/min; Isocratic mobile phase conditions: 75:25 A/B for 30 min. The peak for **5** was observed at a retention time (*t*_R_) of approximately 17 min. The isolated compound was recovered from the eluent via rotary evaporation (Buchi R-114 Rotovapor) and lyophilization (FTS System benchtop lyophilizer). Purity of the final product was 91% as confirmed by LC-CAD. Approximately 1 mg of **5** was provided for spectroscopic and spectrometric analyses.

#### 3.1.2. Nuclear Magnetic Resonance

The sample was prepared by dissolving ~1.0 mg in 180 µL of pyridine-*d*_5_ + TMS, and NMR data were acquired on a Bruker Avance 500 MHz instrument with either a 2.5 mm inverse probe or a 5 mm broad band probe. The ^13^C- and HMBC-NMR data were acquired at Rensselaer Polytechnic Institute using their Bruker Avance 600 MHz and 800 MHz instruments with 5 mm cryo-probe, respectively. The ^1^H- and ^13^C-NMR spectra were referenced to the TMS resonance (δ_H_ 0.00 ppm and δ_C_ 0.0 ppm).

#### 3.1.3. Mass Spectrometry

MS and MS/MS data were generated with a Waters Quadrupole Time-of-Flight Micro mass spectrometer equipped with an electrospray ionization source. The sample was analyzed by negative ESI. The sample was diluted to a concentration of 0.25 mg/mL with H_2_O:MeCN (1:1) and introduced via flow injection for MS data acquisition. The sample was diluted further to 0.01 mg/mL to yield good s/n to tune for MS/MS and acquired by direct infusion. The collision energy was set to 60 V in order to acquire MS/MS data with increased fragment ion peaks due to the nature of the molecule.

### 3.2. Bioconversion Reaction

Rebaudioside I (**5**) was isolated from bioconversion reaction of rebaudioside A (**4**) by a proprietary glucosyltransferase from PureCircle Ltd. *In vivo* production of glycosylation enzymes were expressed in *E. coli*. Rebaudioside A to rebaudioside I conversion with glucosyltransferase UGT76G1-R11-F12 experimental condition are as follows: the reaction mixture (40 mL) contained 0.5 mM rebaudioside A, 3 mM MgCl_2_, 50 mM sodium phosphate buffer at pH 7.5, 2.5 mM UDP-glucose, and 4.0 mL of UGT76G1-R11-F12 (2.5 U/mL). The reaction was run at 30 °C on an orbitary shaker at 135 rpm. For sampling 125 μL of the reaction mixture was quenched with 10 μL of 2N H_2_SO_4_ and 115 μL of methanol/water (7/3). The samples were immediately centrifuged and kept at 10 °C before analysis by by LC-MS. An Agilent 1200 series HPLC system, equipped with binary pump (G1312B), autosampler (G1367D), thermostatted column compartment (G1316B), DAD detector (G1315C), connected with Agilent 6110A MSD, and interfaced with “LC/MSD Chemstation” software, was used. After 42 h. of reaction, 20 mL of the reaction mixture was quenched with 20 mL of ethanol and used for isolation and structure elucidation.

HPLC analyses of samples were performed using an Agilent 1200 series HPLC system equipped with a binary pump (G1312B), autosampler (G1367D), thermostatted compartment (G13136B) and DAD detector (G1315C), connected with Agilent 6110 A MSD, and interfaced with “LC/MSD Chemstation” software. The conditions used were Phenomenex Kinetex, 2.6 μm C18 100A, 4.6 mm × 150 mm, 2.6 μm; Column Temp: 55 °C; Mobile Phase A: 0.1% formic acid in water; Mobile Phase B: Acetonitrile (MeCN); Flow Rate: 0.8 mL/min; Injection volume: 2 µL. Detection was by DAD (210 nm) and MSD (Scan and SIM mode, ES-API, negative polarity). Gradient: 0–8.5 min (76A:24B), 10 min (71A:29B), 16.5 min (70A:30B). SIM parameters 0–4.0 min (SIM ion 1289–1291), 4–11 min (SIM ion 1127–1290), 11–22 min (SIM ion 965–967).

## 4. Conclusions

To our best knowledge this is the first report of full isolation and spectral characterization of (13-[(2-*O*-β-d-glucopyranosyl-3-*O*-β-d-glucopyranosyl)-β-d-glucopyranosyl)oxy]-*ent*-kaur-16-en-19-oic acid-(3-*O*-β-d-glucopyranosyl)-β-d-glucopyranosyl), ester] (rebaudioside I, **5**), from a bioconversion reaction of rebaudioside A. By employing 1D and 2D NMR spectroscopy (^1^H, ^13^C, COSY, HSQC-DEPT, HMBC, 1D TOCSY and NOESY) and mass spectral data, we have completed the full structure elucidation of rebaudioside I. The conversion of rebaudioside A to rebaudioside I demonstrated the power of biotechnology to synthesize a novel steviol glycoside. With continued progress of isolation and characterization of novel steviol glycosides, further relationships between the amount of sugar units connected at C-13 and C-19 of the steviol aglycone core and degree of sweetness will be uncovered.

## Supplementary Materials

Supplementary materials can be accessed at: http://www.mdpi.com/1420-3049/19/11/17345/s1.

## Supplementary Files

Supplementary File 1
