# Supplementary material for: Bioconversion of Rebaudioside I from Rebaudioside A
Source: Molecules. 2014 Oct 28;19(11):17345–55. doi: 10.3390/molecules191117345 (PMC6271207; doi:10.3390/molecules191117345)
Supplement: Supplementary File 1 [file molecules-19-17345-s001.pdf]

Supplementary Materials

Figure S1. ^{1}H-NMR spectrum of **5** at 300 K.

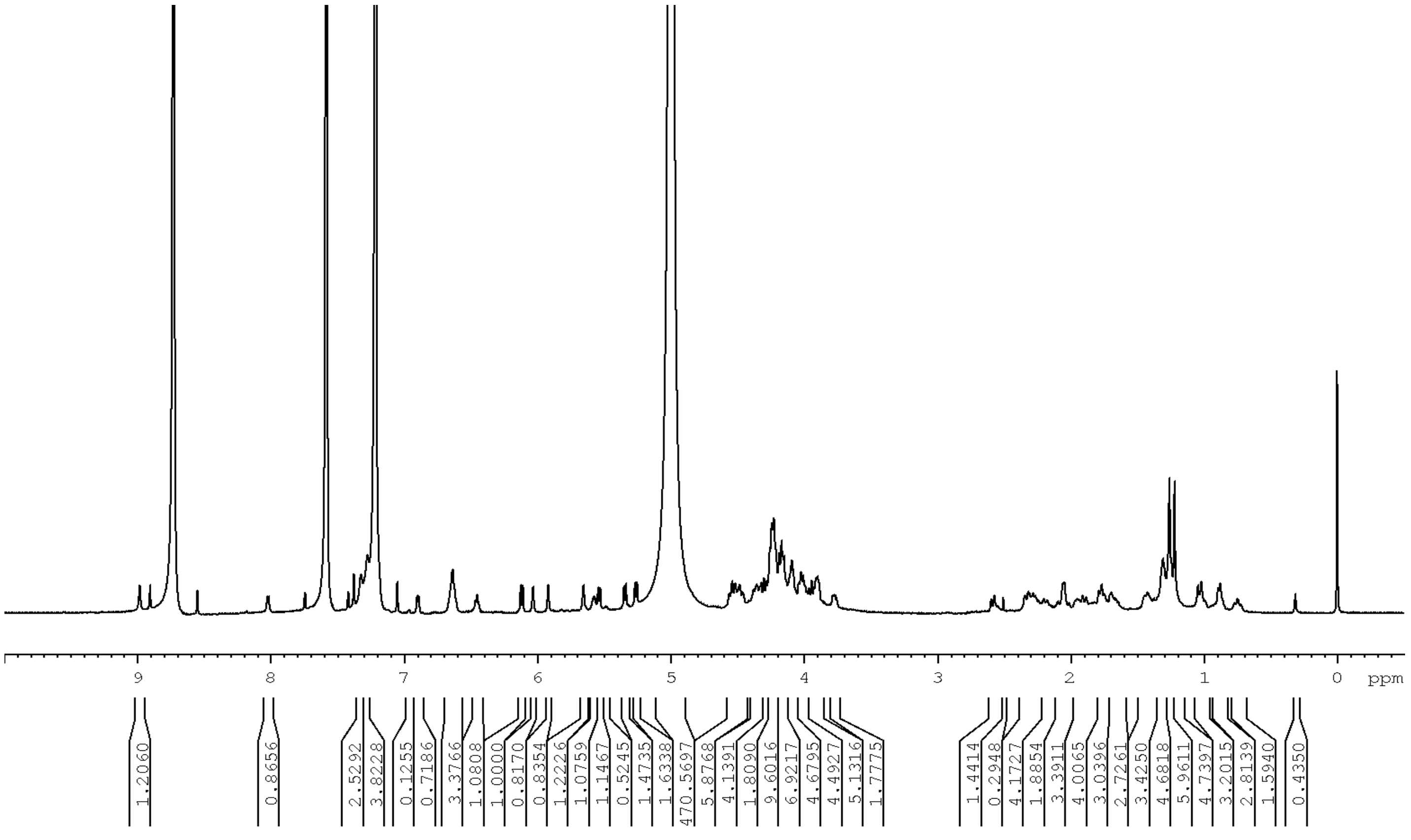

Figure S2. Expansion of ^{1}H-NMR spectrum of **5** at 300 K.

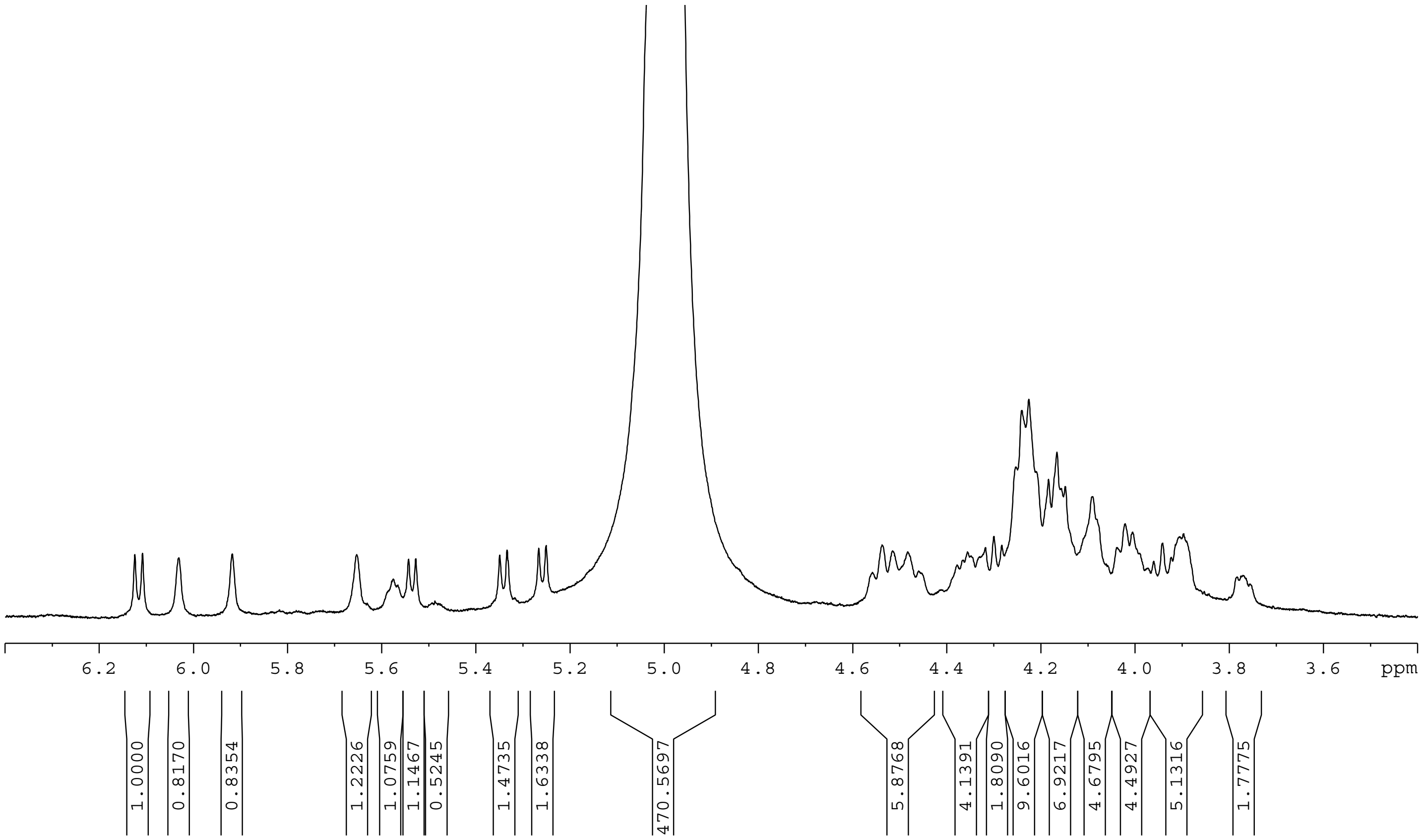

Figure S3. ^{1}H-NMR spectrum of **5** at 292 K.

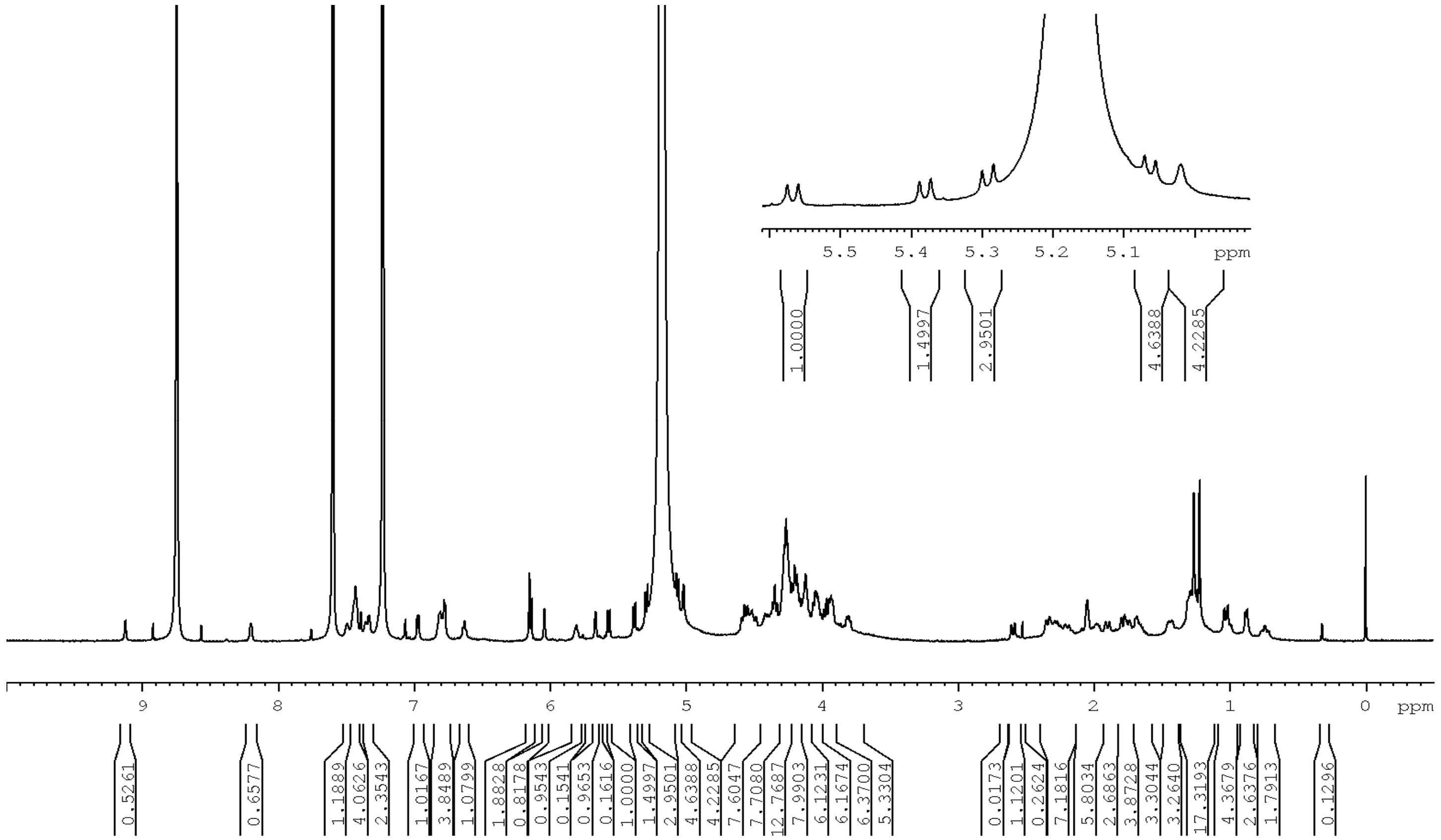

Figure S4. Expansion of ^{1}H-NMR spectrum of **5** at 292 K.

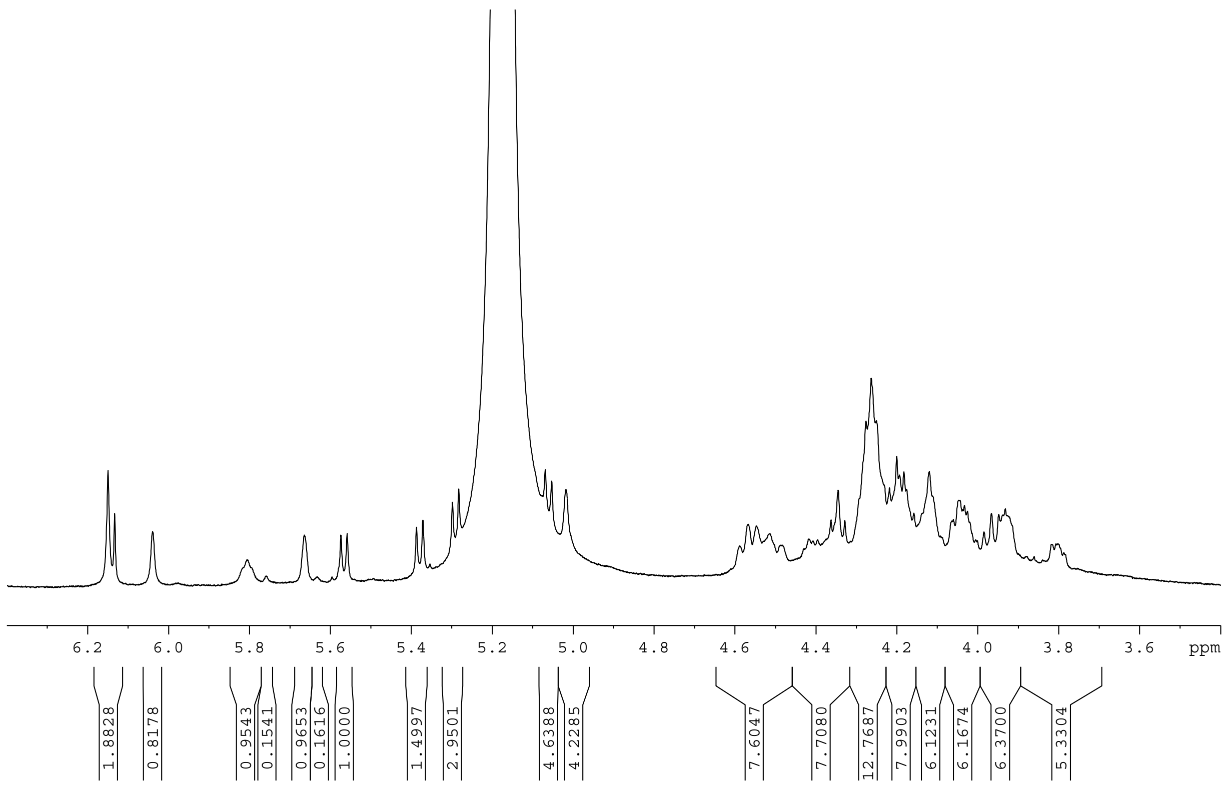

Figure S5. ^{13}C-NMR spectrum of **5** at 292 K.

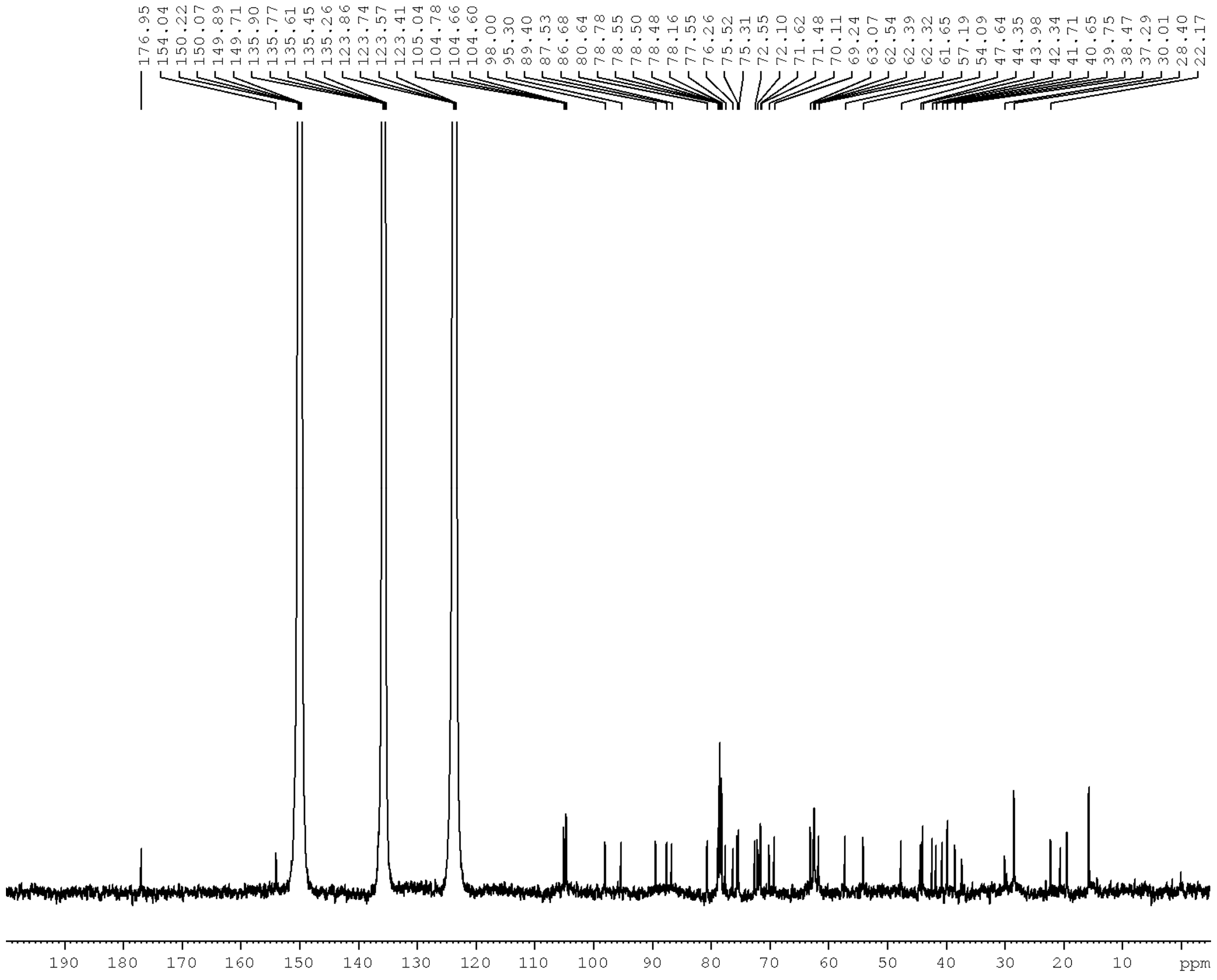

Figure S6. Expansion of ^{13}C-NMR spectrum of **5** at 292 K.

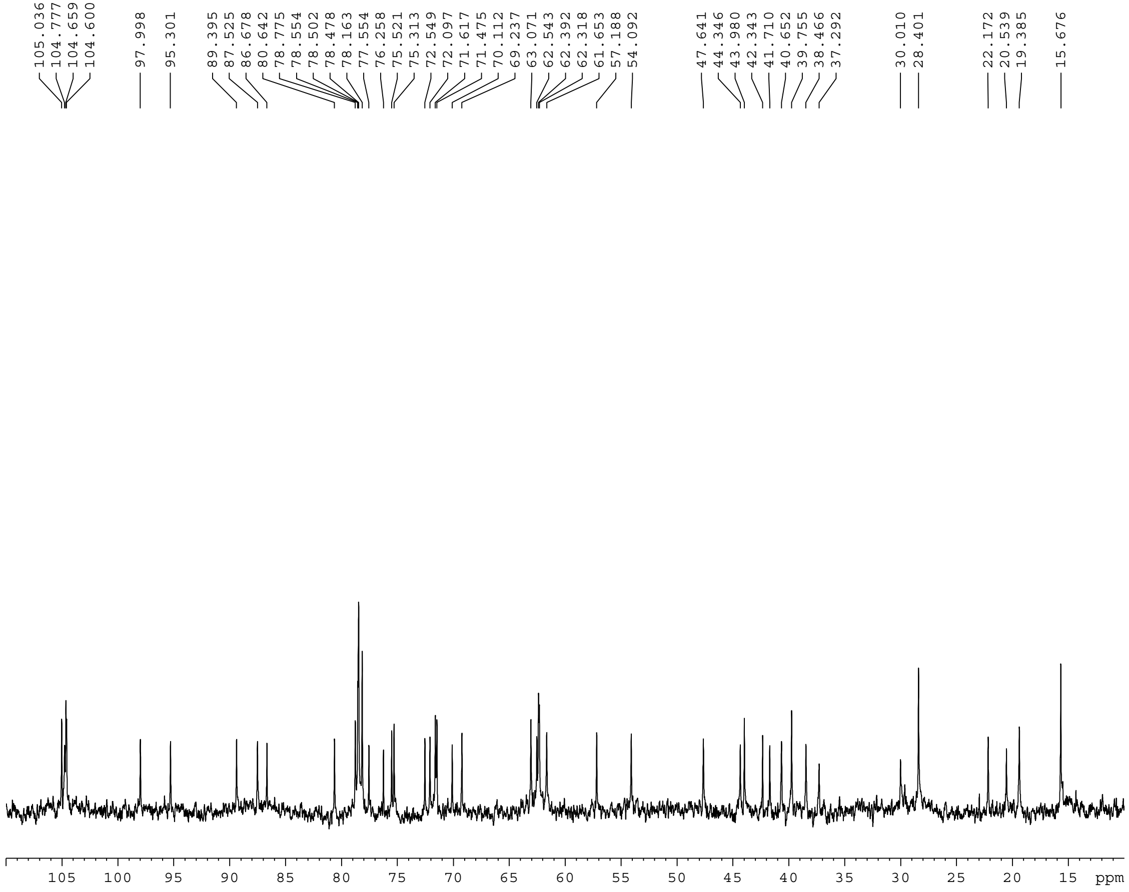

Figure S7. ^{1}H-^{1}H COSY spectrum of **5** at 292 K.

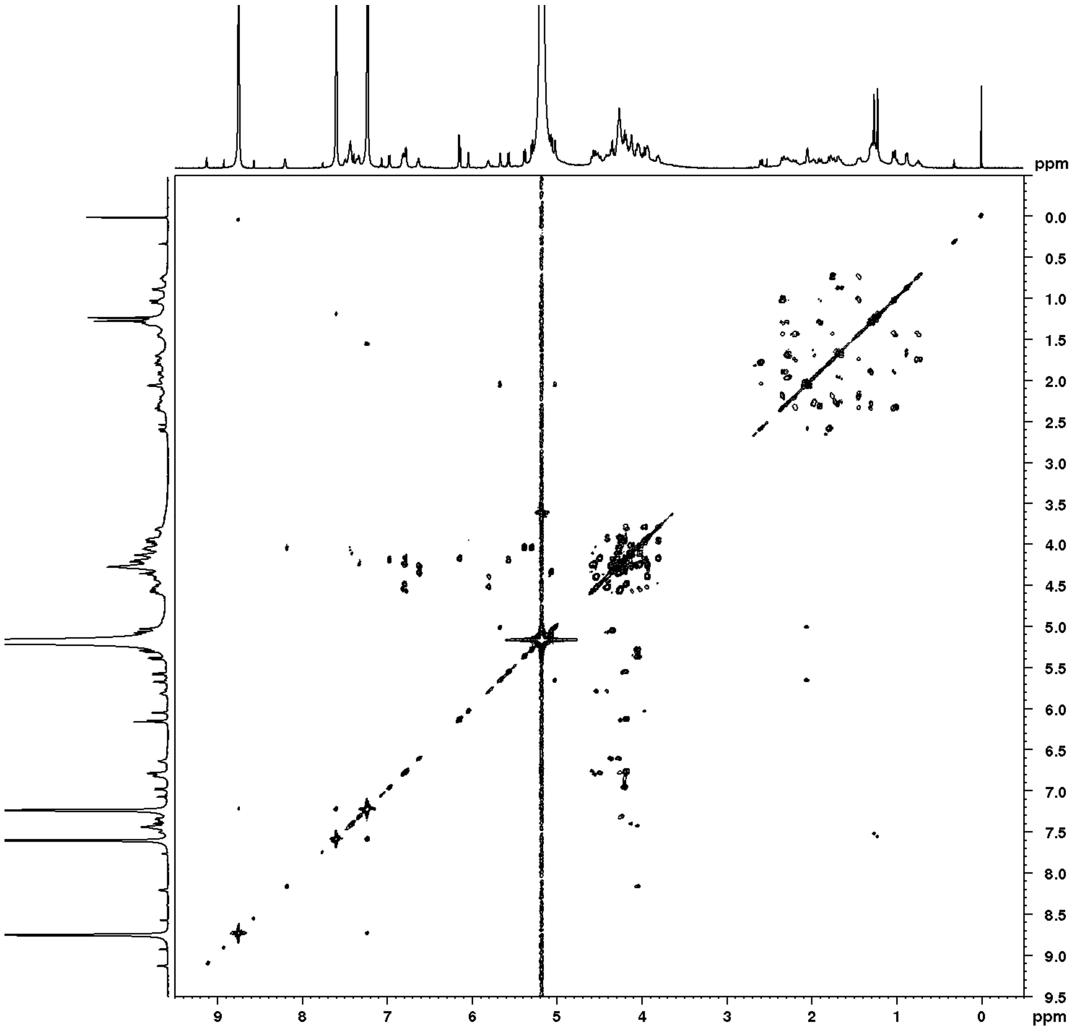

Figure S8. ^{1}H-^{1}H COSY spectrum of **5** at 292 K.

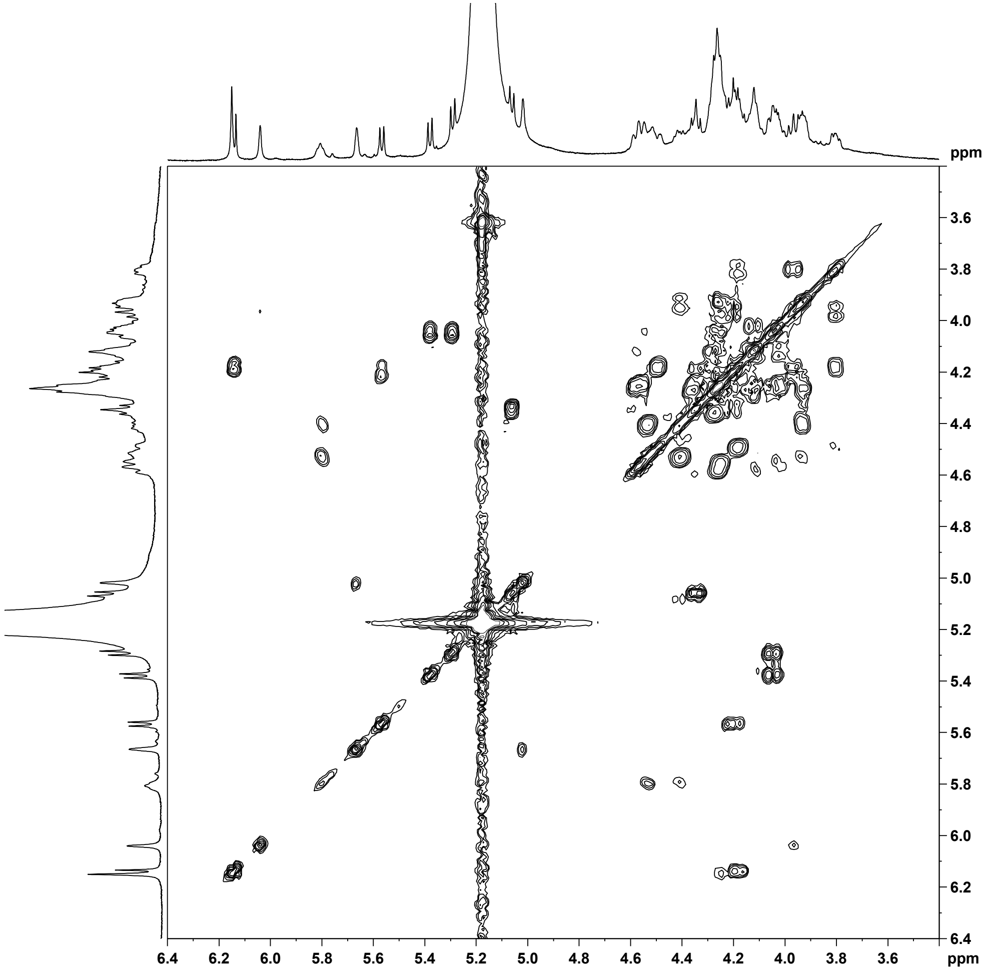

Figure S9. ^{1}H-^{13}C HSQC-DEPT spectrum of **5** at 292 K.

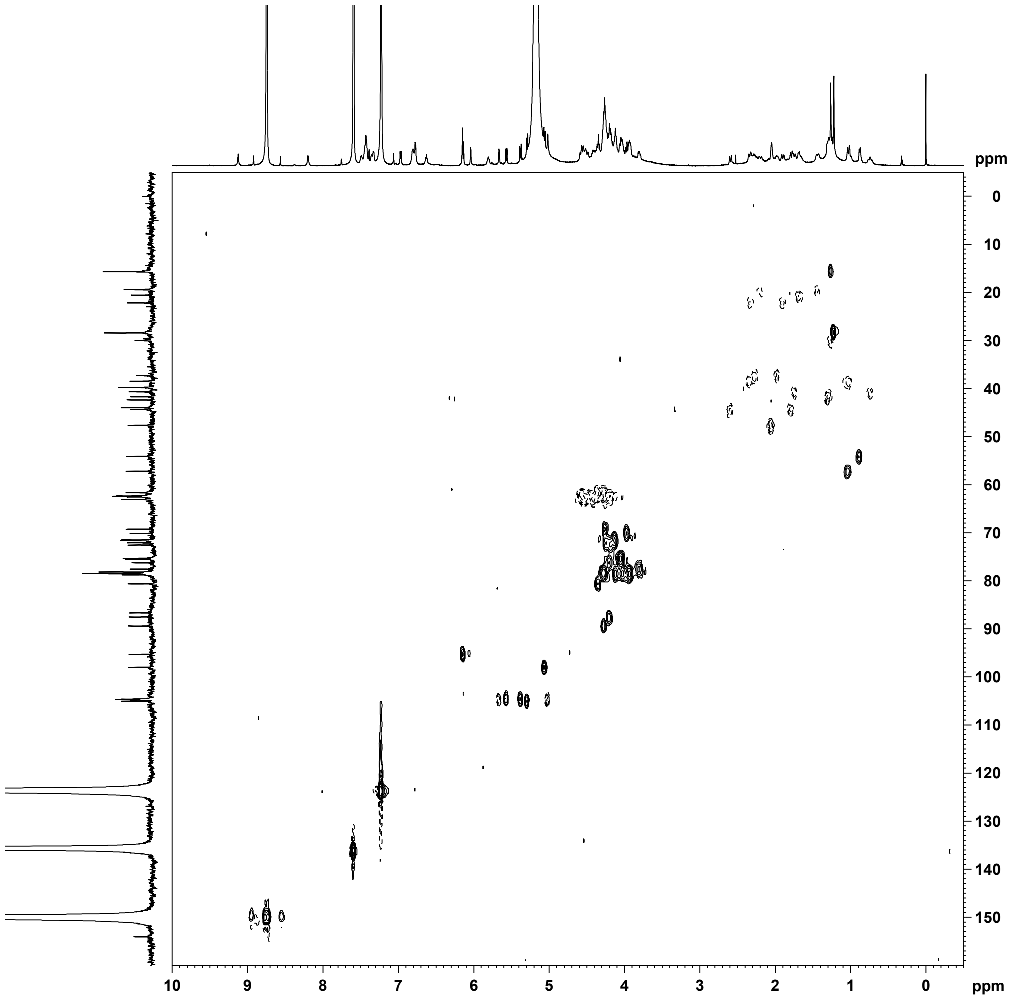

Figure S10. ¹H-¹³C HSQC-DEPT spectrum of **5** at 292 K.

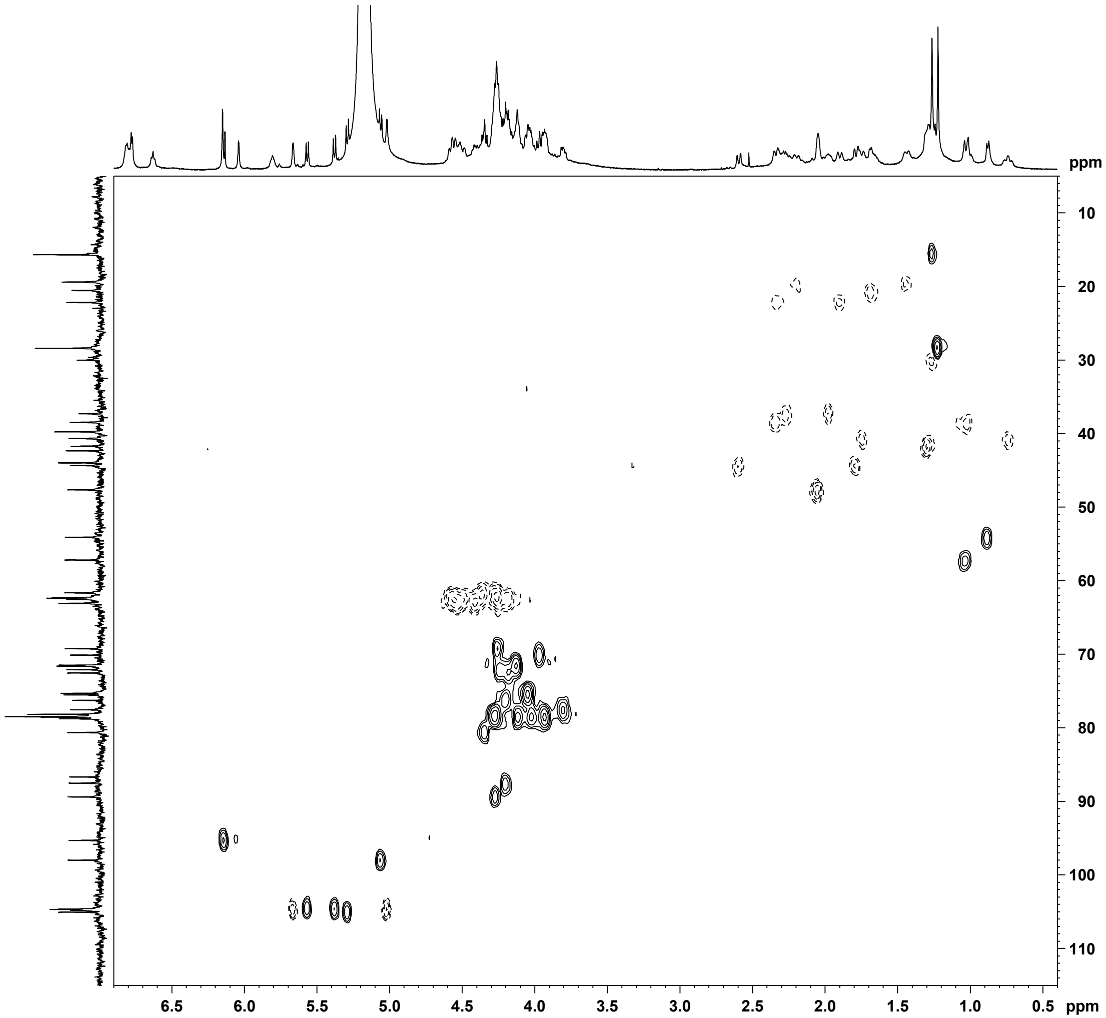

Figure S11. ^{1}H-^{13}C HMBC spectrum of **5** at 292 K.

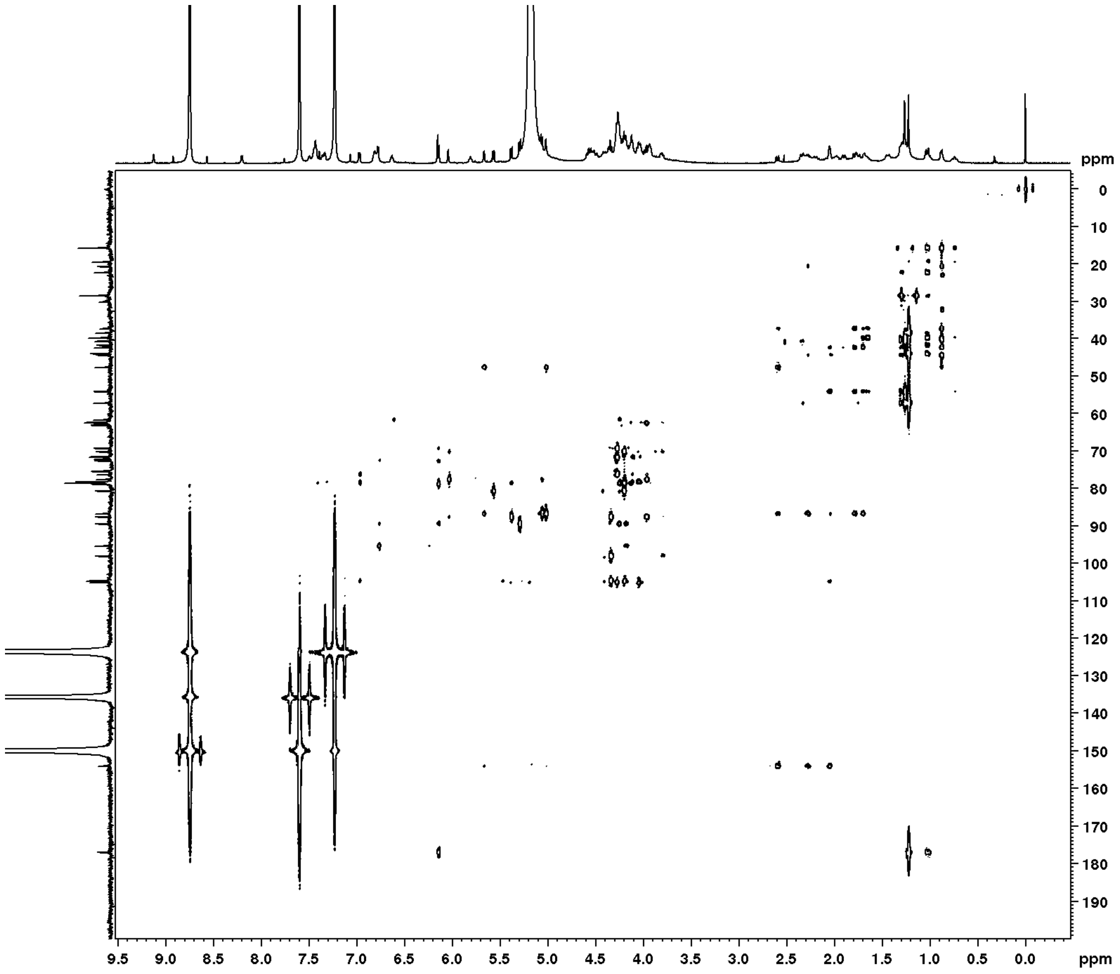

Figure S12. ^{1}H-^{13}C HMBC spectrum of **5** at 292 K.

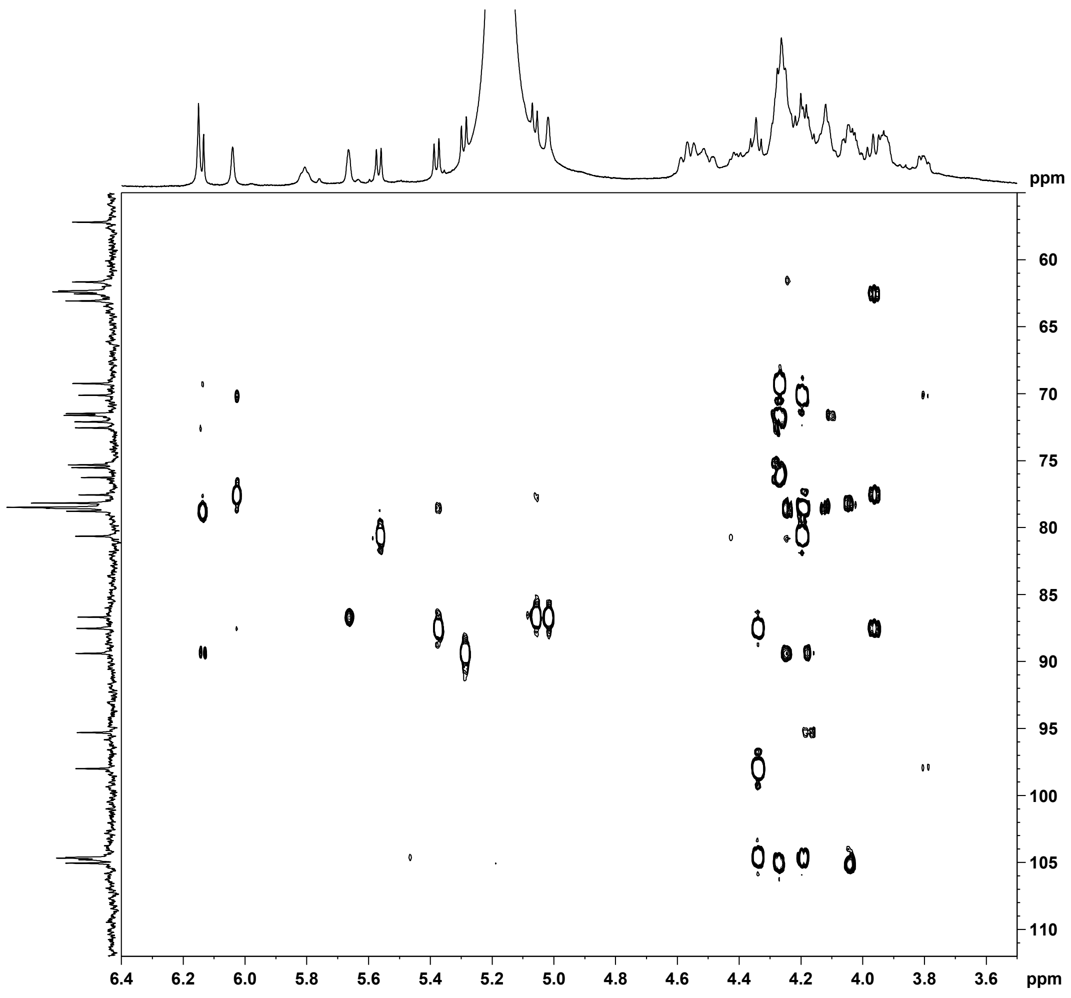

Figure S13. ^{1}H-^{1}H NOESY spectrum of **5** at 292 K.

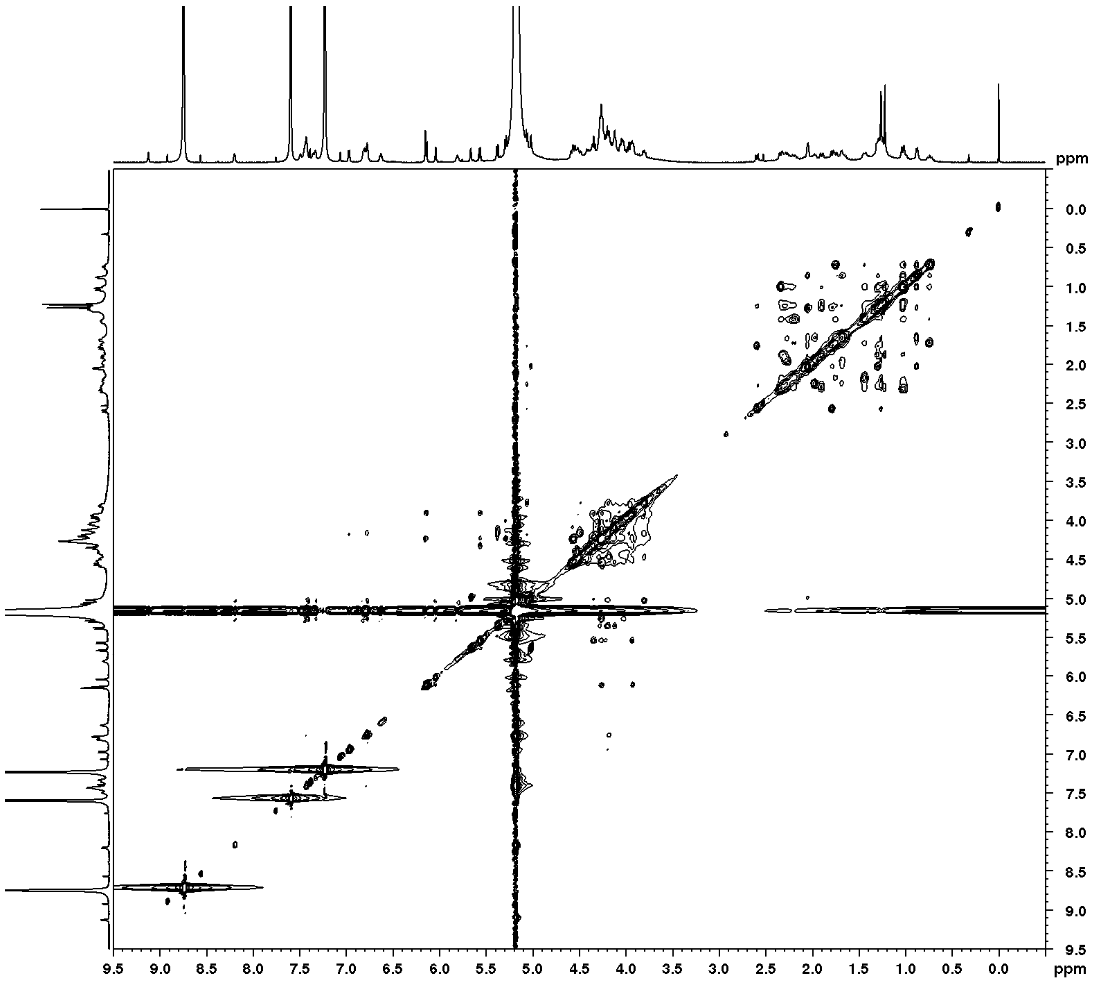

Figure S14. ^{1}H-^{1}H NOESY spectrum of **5** at 292 K.

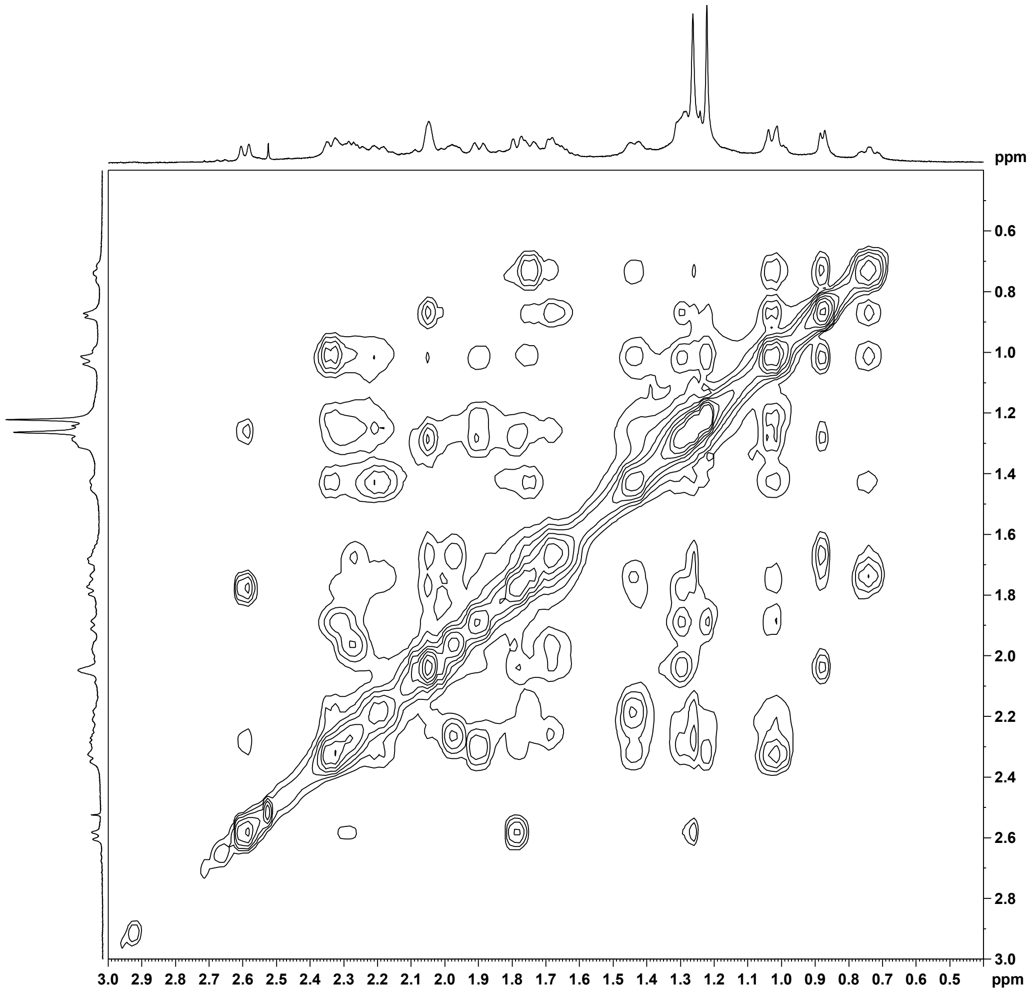

Figure S15. ESI-TOF mass spectrum of Rebaudioside (**5**).

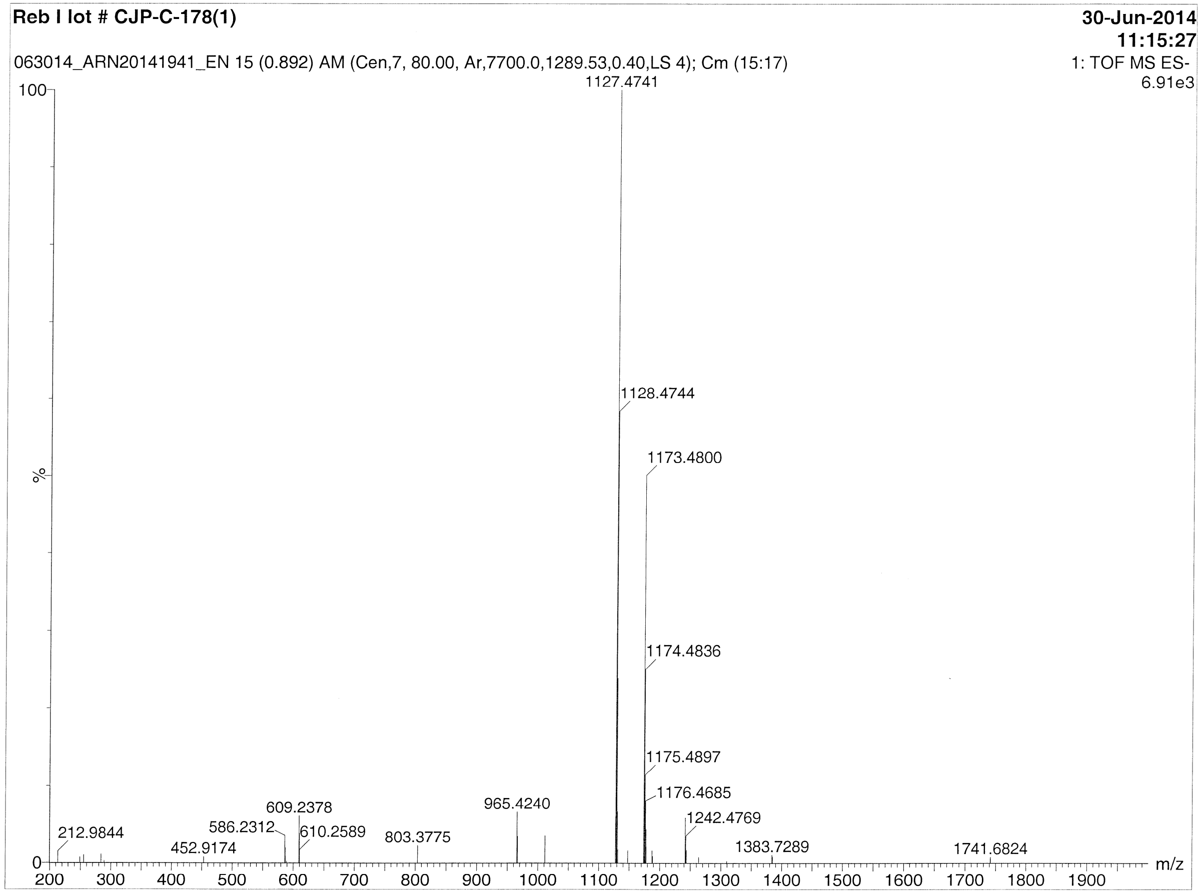

Figure S16. Accurate mass analysis of Rebaudioside (**5**).

Elemental Composition Report

Page 1

Single Mass Analysis (displaying only valid results)

Tolerance = 5.0 PPM / DBE: min = 0.0, max = 50.0
Selected filters: None

Monoisotopic Mass, Even Electron Ions
112 formula(e) evaluated with 2 results within limits (up to 50 closest results for each mass)
Elements Used:
C: 30-60 H: 30-90 O: 0-40

Reb I lot # CJP-C-178(1)

30-Jun-2014
11:15:27

063014_ARN20141941_EN 15 (0.892) AM (Cen,7, 80.00, Ar,7700.0,1289.53,0.40,LS 4); Cm (15:17)

1: TOF MS ES-
6.91e3

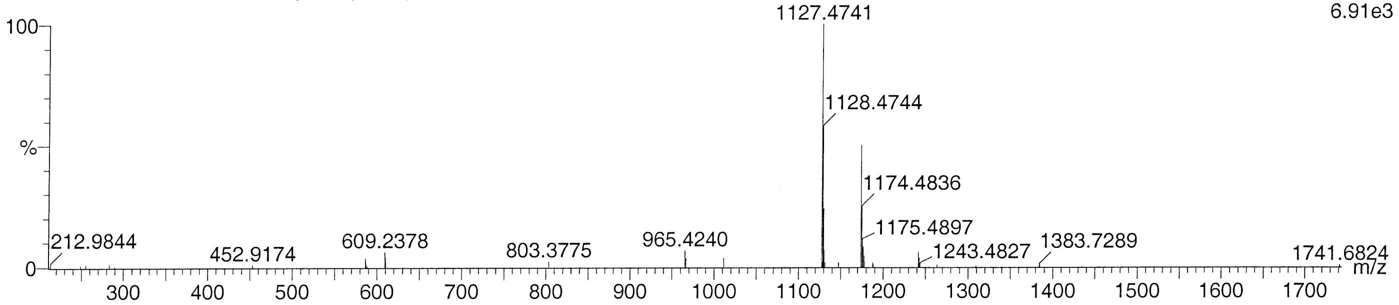

Minimum:				0.0		
Maximum:		100.0	5.0	50.0		
Mass	Calc. Mass	mDa	PPM	DBE	i-FIT	Formula
1127.4741	1127.4758	-1.7	-1.5	11.5	4.5	C50 H79 O28
	1127.4699	4.2	3.7	20.5	17.7	C57 H75 O23
